# Virtual Hospitals and Patient Experience: Protocol for a Mixed Methods Observational Study

**DOI:** 10.2196/58683

**Published:** 2024-10-29

**Authors:** Tim Michael Jackson, Kanesha Ward, Shannon Saad, Sarah J White, Shila Poudel, Freya Raffan, Sue Amanatidis, Jenna Bartyn, Owen Hutchings, Enrico Coiera, Kevin Chan, Annie Y S Lau

**Affiliations:** 1 Centre for Health Informatics, Australian Institute of Health Innovation Macquarie University Sydney Australia; 2 RPA Virtual Hospital (rpavirtual), Sydney Local Health District Sydney Australia; 3 Centre for Social Impact, University of New South Wales Sydney Australia

**Keywords:** virtual care, patient experience, virtual hospital, mixed method, co-design, barriers, facilitators, virtual services

## Abstract

**Background:**

Virtual care is increasingly incorporated within routine health care settings to improve patient experience and access to care. A patient’s experience encompasses all the interactions an individual has with the health care system. This includes a greater emphasis on actively involving carers in the decisions and activities surrounding a patient’s health care.

**Objective:**

This study aimed to investigate the variety of health care delivery challenges encountered in a virtual hospital and explore potential ways to improve the patient experience.

**Methods:**

Focusing on acute respiratory, this protocol outlines a mixed methods study exploring the patient experience of a virtual hospital in Australia, Royal Prince Alfred Virtual Hospital (rpavirtual). We will use an exploratory mixed methods approach comprising of secondary data analysis, observations, interviews, and co-design focus groups. Participants will include patients, their carers, and health care workers who are involved in the acute respiratory virtual hospital model of care. Together, the data will be triangulated to explore views and experiences of using this model of care, as well as co-designing recommendations for further improvement.

**Results:**

Findings from this study will identify current barriers and facilitators to implementing virtual care, such as work-as-done versus work-as-imagined, equity of care, the role of carers, and patient safety during virtual care. As of August 2024, a total of 25 participants have been interviewed.

**Conclusions:**

This protocol outlines a mixed methods case study on the acute respiratory model of care from Australia’s first virtual hospital, rpavirtual. This study will collect the experiences of patients, carers, and health care workers to co-design a series of recommendations to improve the patient experience.

**International Registered Report Identifier (IRRID):**

DERR1-10.2196/58683

## Introduction

### Overview

Virtual health care has expanded across the globe in the wake of the COVID-19 pandemic and has been incorporated into routine health care settings to provide unparalleled access to care [1,2]. However, the push to adopt virtual services has yet to address the challenges and barriers that have risen during their implementation. This protocol outlines a study to clarify and address challenges to virtual services using patient experience as our benchmark. The context of this study will be the Royal Prince Alfred Virtual Hospital (rpavirtual) acute respiratory model of care. rpavirtual is a virtual hospital located in Sydney Local Health District (SLHD), Australia. The goal of this study is to understand the challenges that influence patient experience and co-design recommendations to improve models of virtual care. This study uses an exploratory mixed methods approach of secondary data analysis, observations, interviews, and co-design focus groups.

The rpavirtual acute respiratory model of care offers virtual health care for patients with mild to moderate respiratory illness which can include community-acquired pneumonia, COVID-19, Asthma, or exacerbation of other chronic lung diseases. Suitable patients are referred through the emergency department or their community general practitioner. These patients are provided with a supply of medication and the rpavirtual wearables pack (which contains an oximeter, thermometer, and patient information documentation). The model of care includes 24/7 nursing support, daily virtual medical reviews by phone or video call, 24/7 email messaging, and other care or monitoring tasks as required. Upon discharge, patients are referred to allied health clinicians as required.

### Background

Virtual hospitals have been implemented globally, such as in the United Kingdom, the United States, Australia, Germany, India, Canada, and Saudi Arabia [3-8]. Virtual hospitals aim to deliver hospital-level care remotely through video conferencing, remote monitoring, digital platforms, and linking hospital services with community health care [1,2]. One of the first virtual hospitals was the Atuline Virtual Hospital, which was registered in the European Union in 2001 and was established by a Finnish company to offer online medical consultations and e-prescriptions [5]. In the United States, the Mercy Hospital Virtual Care Centre has been operating in St. Louis, Missouri since 2015, providing rural patients with access to health care providers and specialists (eg, neurologists, intensive care unit clinicians, and stroke care specialists) [8]. In February 2020, the SLHD launched rpavirtual, the first Australian virtual hospital [9]. The aim of rpavirtual is to support patient flow in the district’s acute hospitals by delivering more care in the community, reduce avoidable emergency department presentations, lower hospital admissions, minimize length of stay, enhance the patient experience of care, and inform the broader adoption of virtual health care in the district. In March 2020, rpavirtual had to quickly pivot its focus and respond to the increasing demand for health care services in the COVID-19 pandemic [3].

There is increasing motivation to incorporate hybrid models of care following the COVID-19 pandemic. Notable successes were identified in certain medical specialties such as internal medicine, psychiatry, preventative medicine, surgery, neurology, dermatology, pediatrics, and infectious diseases [10,11]. In addition, a range of visit types was reported to be appropriate for virtual care, such as chronic condition management, rehabilitation, dermatological concerns, mental health support, maternity and parenting services, or matters where there is a pre-established patient-provider relationship (eg, prescription reviews, established diagnoses, or a lack of complex physical examinations) [10,12,13]. Reported findings, pre– and post–COVID-19 pandemic, also indicate that access to virtual care can reduce the burden on in-person resources, assist patients in avoiding transmissible diseases, and empower patients to take a more active role in their own health care [1,2,14]. These benefits should translate to positive patient safety outcomes, reduced hospitalizations, early discharge, and promotion of greater linkages with primary care services [15].

Patient experience is a broad dimension of patient care that describes the quality of interactions patients have with the health care system, health care workers, services, carers, and virtual care technologies [16]. This is an important dimension for virtual models of care as it indicates the ongoing acceptance and trust a patient has in their health care and the likelihood of successful performance in the long run [7-11]. To improve patient experience, patients and carers need to be actively involved in the decisions surrounding their health care both with individual care and health care service design [17,18]. In this study, authors analyze patient experience with a broad lens to be able to assess patient’s preferences and acceptability of interactions with virtual care services, technology, the virtual hospital, health care workers, and carers [1,2]. This metric has become more pressing with the changing environment of health care in Australia. Initial studies report higher levels of patient satisfaction and a willingness to use virtual care again in the future in comparison with earlier virtual care studies pre–COVID-19 pandemic [19]. For example, the study by Manski-Nankervis et al [20] reported that 94% (469/499) of participants found telehealth as an acceptable way to receive health care services in a primary care setting and 97% (485/499) of participants responded that they were comfortable using telehealth technologies.

Despite the great strides made in virtual hospital development, there are still barriers to effectively integrating virtual care within routine health care settings [21-29]. Many challenges identified often stem from the patient experience, such as digital literacy [29], technological difficulties [21-23], unintended burdens on patients [24-26], unintended impact on their carers [25,26], patient safety concerns [29], and questions on privacy and how to ensure confidentiality [22].

There have been studies that have proposed methods of addressing barriers and challenges of video consultations following the COVID-19 pandemic [30,31]. However, few studies have examined the patient experience of virtual hospitals or investigated whether patients are appropriately supported during virtual care [22,26,28]. To explore this area, this study includes the perspectives of health care workers and carers with the aim of understanding their contribution to the patient experience. To summarize, we will investigate patient, carer, and health care worker experiences, identify the challenges of virtual care, and co-design recommendations to improve the patient experience of rpavirtual’s acute respiratory model of care.

### Aim

This study aims to understand and improve the patient experience of the rpavirtual acute respiratory model of care.

### Objectives

First, identify the current patient experience of rpavirtual’s acute respiratory model of care using secondary data and primary observations.

Second, identify the challenges that currently influence the patient experience during virtual care by observing and interviewing a range of stakeholder groups. This includes patients, carers, and health care workers.

Third, co-design a concise, practical, and realistic list of recommendations to mitigate any challenges from engaging with rpavirtual to improve the patient experience of the acute respiratory model of care.

## Methods

### Study Setting

The setting of the study will be a virtual hospital based in Sydney, Australia (rpavirtual). This is the first Australian virtual hospital [9], located in the SLHD. The SLHD is an area of Sydney that contains 5 hospitals, 4 large community health centers, and 12,000 staff members and is responsible for the health of over 700,000 individuals [32]. The rpavirtual acute respiratory model of care provides patients with acute respiratory illnesses with at-home medical devices (eg, pulse oximeter and digital thermometer) to enable remote monitoring and access to hospital support in the home [4,9].

### Study Design

**Figure 1:**
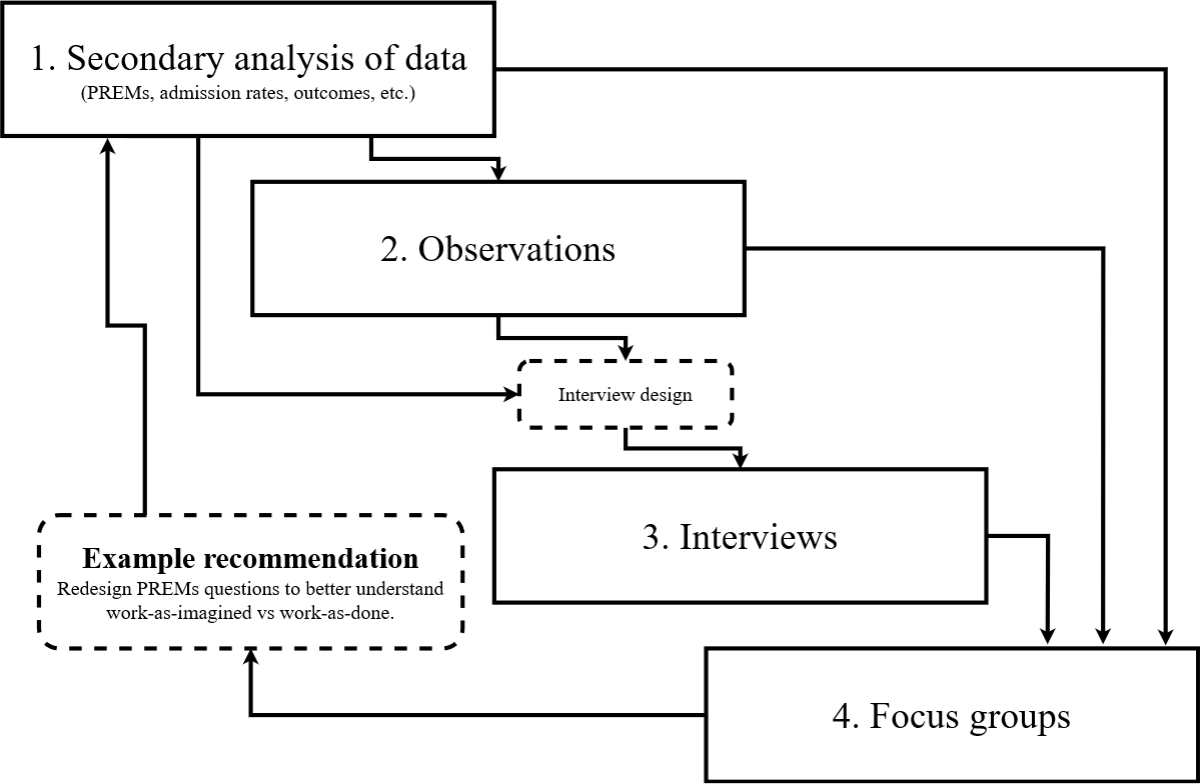
Study process diagram. PREMs: patient-reported experience measures.

This study will use an exploratory mixed methods approach to explore patient experience using virtual care. The structure will comprise of four stages, that are (1) analyzing existing patient data, (2) observing virtual consultations, (3) interviewing participants, and (4) holding focus groups (Figure 1).

Considering the innovative nature and relative novelty of virtual hospitals, we designed a comprehensive method, drawing on existing approaches to investigate new phenomena [33,34], explore complex systems [35], and gain a holistic understanding of an event or interaction [36]. The aim is to produce a practical list of recommendations for improvement founded on action-based evidence [37].

Research activities of secondary analysis of routinely collected data, observations, and interview activities shall run simultaneously (ie, insights from different stages may inform other activities simultaneously). The interviews shall follow a semistructured design to adapt to insights from other research stages while upholding participant anonymization. Microsoft Teams will be used to transcribe observation recordings and interviews with participants. The accuracy of the transcription is checked by authors KW and AL.

#### Stage 1: Secondary Data Analysis

The initial stage of this research study is the analysis of secondary data collected from standard hospital procedures as part of the acute respiratory model of care. This is the preliminary stage of the mixed methods design to understand complex systems through a quantitative foundation [35]. The data used for analysis will include patient-reported experience measures (PREMs), readmission rates, length of stay, mortality, and adverse outcomes. Descriptive statistical analysis on patient demographics and patient experience will be conducted, using deidentified electronic medical records (eMRs) and anonymized PREMs data sets. PREMs survey responses will be tested using standard statistical tests (including but not limited to factor analysis) to identify potential relationships between participant groups and their responses. This data will also be used to inform the study design of prospective data collection approaches (observations, interviews, and focus groups), and in triangulation of data analysis with the other research activities.

#### Stage 2: Consultation Observations

The second stage involves observations of current practices in the acute respiratory model of care at rpavirtual. This is the first qualitative stage of the mixed methods design with the aim of understanding the current process of providing virtual care [36]. The observations will take place in virtual consultations with patients. The audio and video will be recorded using external audio devices or Microsoft Teams and field notes will be created.

Content analysis and other qualitative data analysis approaches will be used to understand the patient experience of rpavirtual (eg, thematic analysis, conversation analysis, and linguistic ethnography). The type and range of data analysis approaches will be subject to the sample size and variances of observations we are able to obtain during the data collection of the research study. We will explore how information is communicated between clinician, patient, and carer (eg, how patients and carers notify clinicians of results collected from patient-facing digital devices such as an oximeter during virtual consultations). Quantitative descriptive statistical tests will also be used to compare consultations with each other and the literature (eg, length of consultation and frequency of mentioning use of patient-facing digital devices during consultation). This data will also be used in triangulation with the other research activities.

#### Stage 3: Interviews

The third stage is a series of interviews with individuals involved in the acute respiratory model of care. This includes health care workers, patients, and their carers. The aim of this stage is to explore known challenges identified from the perspectives of health care workers, patients, and carers [38]. The interviews will last from 30 to 40 minutes. Video and audio will be recorded using an external audio device and Microsoft Teams and transcribed verbatim. Unknown challenges will be identified and analyzed using an inductive approach, where we will collect patient experience perspectives through interviews and narratively analyze the data to make sense of the individual participants’ responses. The qualitative methods of constant comparison and axial coding shall be used to identify the relationships and themes between responses, comparing similarities and differences between responses to sort the data [39]. Then, we will explore any issues unraveled to improve the patient experience. Individual biases will be addressed by triangulating different sources of data (ie, deidentified routinely collected data from rpavirtual, observations made by researchers, and interview self-reported data by research participants). Using multiple methods and sources help counteract biases inherent in any single approach, leading to more objective conclusions. Multiple researchers will be involved in the data coding and analysis stages to reduce the impact of individual biases.

#### Stage 4: Co-Design Focus Groups

The fourth and final stage of this research is a series of focus groups. Focus groups are a useful method to explore the experiences, attitudes, and challenges of different stakeholder groups [40]. They achieve this by emphasizing group interaction and fostering group discussion of ideas [41]. We will follow the co-design framework of Boyd et al [42], which used patient journey mapping, experience-based surveys, and co-design workshops to improve the patient experience. We have also included the modification suggested by Yeates et al [43] of including a refinement stage [43]. We will undertake a collaborative approach, conducting a series of focus group workshops to delve deeper into developing solutions to barriers and challenges identified.

Preliminary findings from the previous stages of the study will be explored in the focus groups. We will intentionally involve participant groups in designing solutions and recommendations through a participatory approach. We will gather feedback, synthesize feedback into insights using a thematic and comparative analysis, and develop solutions and recommendations based on that feedback.

The aim here is to co-design concise and realistic recommendations to improve the patient experience. Each participant group (health care workers, patients, and carers) will have a focus group to discuss potential challenges they have encountered. A final focus group, using a combination of individuals from different participant groups, will synthesize the key points from the previous groups. All focus groups will range from 50 to 60 minutes. Audio will be recorded and transcribed verbatim.

### Participants

A total of 3 participant groups have been identified in this study. First, health care workers—any worker involved in the virtual care of patients under the acute respiratory model of care at rpavirtual. This includes medical doctors, nurses, technical support staff, and allied health staff. Second, patients—any individual who received treatment for an acute respiratory illness. Third, carers—individuals who provide support and care for a patient who has received care under the acute respiratory model of care at the virtual hospital. These could be formal (ie, someone employed to provide a professional carer service such as a paid nurse) or informal relationships (ie, someone not employed to provide carer support such as a family member).

Participant numbers have yet to be determined as it is limited to patients admitted to the acute respiratory model of care. The goal of a qualitative study is to reach saturation of knowledge, where relevant, as well as methodology-specific indicators for validity [44]. The saturation of knowledge is based on the level of information gained and triangulated from all research activities, including observations, interviews, focus groups, and PREM survey open-ended responses [45]. In a mixed methods study using qualitative interviews and observations, we shall recruit participants until we reach data saturation, which is often around 5 to 15 participants according to qualitative studies in similar areas [46,47]. Considering the various participant groups, informed by general guides from previous studies, the aim will be for 5 or more individuals from each participant group (health care workers, patients, and carers) but researchers will aim for as many as possible from each group until we reach data saturation [48].

### Participant Recruitment

Potential participants will be identified by rpavirtual. Patients will first be asked by their health care worker if they are interested in possibly participating, and if they consent, the research team will share the recruitment material. Patients will be encouraged to ask their carers to participate in the research study, to which if they consent, the REDCap (Research Electronic Data Capture; Vanderbilt University) e-consent form will be forwarded to those patients and carers. The research team does not have access to patient records of the rpavirtual. To motivate patients and carers, a financial gratuity will be offered. Any patient or carers who participate in an interview will receive a gift card of AUD $40 (US $26.71) and if they participate in a focus group will receive a further AUD $60 (US $40.06). A currency exchange rate of AUD $1=US $0.67 is applicable as of September 3, 2024. Financial gratuity will not be offered for observations to reduce potential Hawthorne effect and subject bias. Patients’ clinical care will not be impacted by their choice to participate (or not) in consultation observations. Health care worker staff members will be recruited through internally shared promotional videos, internal education sessions and presentations, and word of mouth.

### Consent Process

In addition to obtaining written consent or e-consent, participants will be asked for verbal consent before involvement in any observation, interview, and focus group activity. Consent will be requested at multiple stages and at each stage the participant will be reminded that they can withdraw their consent and exit the study at any time. They will also be informed that withdrawing from the study will have no impact on their employment, the health care provided to them, or their relationship with the research team and the universities conducting the study. Participants will have the option to consent to each individual research activity (eg, observations, interviews, and focus groups). Only currently admitted patients may be included in the consultation observations. Current or discharged patients may be included in interviews and focus groups. Email and SMS reminders will be sent to participants (up to 2 times over 2-4 weeks). Consenting discharged patients may participate in interviews and focus group activities. In the scenario when e-consent cannot be elicited before a consultation, verbal consent from all parties may be elicited during the consultation and before recording the consultation. The research team will continue to elicit e-consent from all parties involved after the consultation recording. Therefore, discharged patients may complete an e-consent form for the research team getting access to their consultation recordings, which were collected and recorded with their verbal consent while being admitted. If there is no response after following up, researchers will assume that the individual does not wish to participate and will cease follow-up contact.

#### Step 1: Identification of Potential Participants

The research team at Macquarie University (MQ) will provide study material, the participant information consent form, and advertisement material to rpavirtual to be disseminated to potential participating health care workers, patients, and carers of consenting health care workers, seeking permission for MQ researchers to contact them. MQ researchers will then initiate contact with the potential participant using their preferred contact mechanism, or at their next interaction with the participating virtual hospital clinician.

#### Step 2: First Contact With Potential Participant

Initial contact may occur in the following ways: participant reaches out to MQ researchers for more information, MQ researcher contacts potential participants through their preferred contact channel after they have consented to their health care worker, or during their virtual consultation session to explain the study and elicit their informed consent (written and verbal) after the patients have expressed interest through their health care workers.

#### Step 3: Further Information Given

Potential participants will be provided the participant information packet and REDCap e-consent form through SMS or email or web link. Once the study promotion material is disseminated, the contact point will be MQ researchers. Potential participants are to be provided an opportunity to ask questions about the study either by email, telephone, or face-to-face before signing a consent form.

#### Step 4: Return of Consent Forms

Participant returns signed REDCap e-consent form. If no response, researchers will follow up with the participant (up to 2 times over 2-4 weeks). In the scenario when e-consent was not elicited before recording a consultation, the research team will continue to elicit e-consent from all parties involved after the consultation recording before gaining access to conduct analysis. If no response after following up, researchers will assume the individual does not wish to participate and cease follow-up contact.

### Privacy and Data Security

Electronic consent forms will be stored on REDCap using the SLHD license. Electronic consent forms will be securely archived in the study REDCap project. Hard copies of written consent forms will be securely stored at rpavirtual Research and Evaluation Hub in a locked filing cabinet only accessible to the research team.

We will use the following methods to protect the data collected. Access restrictions (physical key lock and passwords), deidentification (anonymized data), and storing on institutional networks at rpavirtual and MQ. Deidentified eMR and anonymized PREMs data will be stored securely in MQ Microsoft Teams and deleted 5 years after analysis is completed. External audio devices and Microsoft Teams will be used to record observations, interviews, and focus groups. Personal mobile devices will not be used for audio recording or video recording. Only investigators in the research team have access to the data collected.

A custom-made software was developed in-house to blur the faces of individuals appearing in consultation videos, ensuring the privacy of participants before analysis.

### Ethical Considerations

#### Human Participants Ethics Review Approvals or Exemptions

This research involves humans, medical records, patient information, observations of public behaviors, or secondary data analyses. This study adheres to appropriate ethical review and approvals, as per institutional guidelines. Ethical approval for this project was obtained from the Macquarie University Human Research Ethics Committee for Medical Sciences (reference 520231595552727) and the Sydney Local Health District Ethics Review Committee (Royal Prince Alfred Hospital Zone; reference 2023/ETH01269). The project has been approved and supported by the rpavirtual Research Steering Committee.

#### Informed Consent

Participants of this research study are provided the opportunity for informed consent. Participants are to be provided the ability to withdraw from the study up until the point of the data being deidentified for analysis. For secondary analyses of routinely collected data (ie, PREMs), original consent was obtained by rpavirtual with the original consent covering secondary analysis without additional consent.

#### Privacy and Confidentiality

Primary data collected (ie, interviews, focus groups, observations, and eMR data) will be stored and analyzed in a deidentified format. Secondary data collected from routinely collected data (ie, PREMs) will be collected, stored, and analyzed in a deidentified and anonymized format.

#### Compensation Details

Any patient or carers who participate in an interview will receive a gift card of AUD $40 (US $26.71) and if they participate in a focus group will receive a further AUD $60 (US $40.06). Health care workers will not receive any financial gratuity for participating in this research study.

## Results

The project has started recruitment of participants for observations and interviews. As of August 2024, over 25 participants have been interviewed. All recorded consultations, field notes, interviews, and focus groups will be recorded and transcribed verbatim. The software NVivo (Lumivero) will be used to manage transcripts and analysis. Triangulation [38], content [49], and thematic analysis will be used to identify commonalities. The analysis of our study shall potentially investigate any or all of the barriers and challenges identified, and the known or unknown solutions from the perspectives of patients, their carers, or health care workers. Open coding will also be used to identify any additional themes not previously uncovered [36]. The context and associated factors of the consultation will be analyzed through linguistic ethnography. Each aspect of patient experience shall be triangulated to provide a comprehensive analysis. We anticipate results to (1) narrate patients experiences with rpavirtual services, the role of patients’ carers, and their interactions with virtual care services; (2) list and detail known challenges, unknown challenges, successes, and types of patient interactions with virtual care; and (3) create realistic and comprehensive solutions to the challenges identified to improve upon the narrated patient experiences with rpavirtual services.

## Discussion

### Principal Findings

This paper outlines an ongoing study investigating patient experiences of using rpavirtual’s virtual hospital services in the acute respiratory model of care, aiming to identify the types of challenges and propose solutions. By using a mixed methods approach of observations, interviews, focus groups, and analysis of routinely collected data, we will provide a comprehensive analysis of the current patient experience and challenges. Our exploratory approach aims to map patients’ journeys, providing case studies of interactions that result in positive patient experiences or may benefit from further support, involving patients’ carers and health care workers. By triangulating insights from these perspectives, we aim to uncover both realized and unrealized challenges, contributing important insights to the current literature, and facilitating the co-design of impactful solutions. The findings of this research will be disseminated to metropolitan, national, and international audiences through publication in peer-reviewed journals and presentations at scientific conferences.

### Strength and Limitations

This study’s strength lies in its mixed method approach (routinely collected PREMs, observations, interviews, and focus groups) with multiple stakeholders (patients, carers, and health care workers) to investigate the patient experience of a virtual hospital. Existing methods of collecting patient experience information (eg, PREMs data) do not consider the perspectives of health care workers or carers. Using a multi-method, staged approach ensures we are capturing a comprehensive picture of patient experience from different perspectives. Furthermore, our research team includes a combination of academics with expertise in health informatics, mixed methods, clinical science, and the involvement of practicing clinicians (nurses and medical doctors) and key staff from the virtual hospital. The diverse, multidisciplinary research team ensures our research investigations are inclusive and relevant and that findings can be directly applied to improve current practices.

This study does not include clinical and medical data of patients. This level of access is not required to understand the patient experience. The risks of such access are outweighed by the benefits generated for the participant; thus, only basic deidentified administrative data of a patient’s admission in the virtual hospital is included in our analysis.

### Conclusion

The findings from this study will contribute significantly to the limited research on patient experiences with virtual hospital services, adding new perspectives by using multiple methods and participant groups. The integration of quantitative and qualitative methods will allow the identification and in-depth analysis of realized and unrealized challenges patients, their carers, and health care workers face. We anticipate that insights from this study will guide the development of targeted solutions to address unmet needs and challenges, facilitating the co-design of practical and meaningful solutions to enhance patient experiences in this health care setting.

## Abbreviations

eMR: electronic medical record
MQ: Macquarie University
PREM: patient-reported experience measure
REDCap: Research Electronic Data Capture
rpavirtual: Royal Prince Alfred Virtual Hospital (a hospital in Sydney, Australia. Part of the SLHD)
SLHD: Sydney Local Health District (a collection of health services and hospitals run by the Australian Government in Sydney, Australia)

## References

[1] Snoswell CL, Caffery LJ, Haydon HM, Thomas EE, Smith AC (2020). Telehealth uptake in general practice as a result of the coronavirus (COVID-19) pandemic. Aust Health Rev.

[2] Johnsen TM, Norberg BL, Kristiansen E, Zanaboni P, Austad B, Krogh FH, Getz L (2021). Suitability of video consultations during the COVID-19 pandemic lockdown: cross-sectional survey among Norwegian general practitioners. J Med Internet Res.

[3] Raffan F, Anderson T, Sinclair T, Shaw M, Amanatidis S, Thapa R, Nilsson SJ, Jagers D, Wilson A, Haigh F (2021). The virtual care experience of patients diagnosed with COVID-19. J Patient Exp.

[4] Mannix L (2020). Australia's first virtual hospital rolls out for COVID-19 patients.

[5] Syrjänen E (2000). Building a virtual hospital.

[6] Khashogji Z Saudi Arabia launches first virtual hospital. Arabia News.

[7] Alkhalifah JM, Seddiq W, Alshehri BF, Alhaluli AH, Alessa MM, Alsulais NM (2022). The role of the COVID-19 pandemic in expediting digital health-care transformation: Saudi Arabia's experience. Inform Med Unlocked.

[8] Bidoli C, Pegoraro V, Dal Mas F, Bagnoli C, Bert F, Bonin M, Butturini G, Cobianchi L, Cordiano C, Minto G, Pilerci C, Stocco P, Zantedeschi M, Campostrini S (2023). Virtual hospitals: the future of the healthcare system? An expert consensus. J Telemed Telecare.

[9] Torres-Robles A, Allison K, Poon SK, Shaw M, Hutchings O, Britton WJ, Wilson A, Baysari M (2023). Patient and clinician perceptions of the pulse oximeter in a remote monitoring setting for COVID-19: qualitative study. J Med Internet Res.

[10] Ward K, Vagholkar S, Sakur F, Khatri NN, Lau AYS (2022). Visit types in primary care with telehealth use during the COVID-19 pandemic: systematic review. JMIR Med Inform.

[11] Doraiswamy S, Abraham A, Mamtani R, Cheema S (2020). Use of telehealth during the COVID-19 pandemic: scoping review. J Med Internet Res.

[12] Gabrielsson-Järhult F, Kjellström S, Josefsson KA (2021). Telemedicine consultations with physicians in Swedish primary care: a mixed methods study of users' experiences and care patterns. Scand J Prim Health Care.

[13] Due TD, Thorsen T, Andersen JH (2021). Use of alternative consultation forms in Danish general practice in the initial phase of the COVID-19 pandemic - a qualitative study. BMC Fam Pract.

[14] Mulinacci G, Alonso GT, Snell-Bergeon JK, Shah VN (2019). Glycemic outcomes with early initiation of continuous glucose monitoring system in recently diagnosed patients with type 1 diabetes. Diabetes Technol Ther.

[15] Moore G, Du Toit A, Jameson B, Liu A, Harris M (2020). The effectiveness of virtual hospital models of care. A Rapid Evidence Scan brokered by the Sax Institute for Sydney Local Health District.

[16] Oben P (2020). Understanding the patient experience: a conceptual framework. J Patient Exp.

[17] Rossiter C, Levett-Jones T, Pich J (2020). The impact of person-centred care on patient safety: an umbrella review of systematic reviews. Int J Nurs Stud.

[18] Cherba M, Grosjean S, Bonneville L, Nahon-Serfaty I, Boileau J, Waldolf R (2022). The essential role of nurses in supporting physical examination in telemedicine: insights from an interaction analysis of postsurgical consultations in orthopedics. Nurs Inq.

[19] Imlach F, McKinlay E, Middleton L, Kennedy J, Pledger M, Russell L, Churchward M, Cumming J, McBride-Henry K (2020). Telehealth consultations in general practice during a pandemic lockdown: survey and interviews on patient experiences and preferences. BMC Fam Pract.

[20] Manski-Nankervis J, Davidson S, Hiscock H, Hallinan C, Ride J, Lingam V, Holman J, Baird A, McKeown E, Sanci L (2022). Primary care consumers’ experiences and opinions of a telehealth consultation delivered via video during the COVID-19 pandemic. Aust J Prim Health.

[21] Garnett A, Northwood M, Ting J, Sangrar R (2022). mHealth interventions to support caregivers of older adults: equity-focused systematic review. JMIR Aging.

[22] Yi JS, Pittman CA, Price CL, Nieman CL, Oh ES (2021). Telemedicine and dementia care: a systematic review of barriers and facilitators. J Am Med Dir Assoc.

[23] Söylemez BA, Özgül E, Küçükgüçlü Ö, Yener G (2023). Telehealth applications used for self-efficacy levels of family caregivers for individuals with dementia: a systematic review and meta-analysis. Geriatr Nurs.

[24] Ferraris G, Dang S, Woodford J, Hagedoorn M (2022). Dyadic interdependence in non-spousal caregiving dyads' wellbeing: a systematic review. Front Psychol.

[25] Gaigher JM, Lacerda IB, Dourado MCN (2022). Dementia and mental health during the COVID-19 pandemic: a systematic review. Front Psychiatry.

[26] Hopwood J, Walker N, McDonagh L, Rait G, Walters K, Iliffe S, Ross J, Davies N (2018). Internet-based interventions aimed at supporting family caregivers of people with dementia: systematic review. J Med Internet Res.

[27] Zhu A, Cao W, Zhou Y, Xie A, Cheng Y, Chu S (2021). Tele-health intervention for carers of dementia patients-a systematic review and meta-analysis of randomized controlled trials. Front Aging Neurosci.

[28] Airola E (2021). Learning and use of eHealth among older adults living at home in rural and nonrural settings: systematic review. J Med Internet Res.

[29] Hyman P (2020). The disappearance of the primary care physical examination-losing touch. JAMA Intern Med.

[30] Seuren LM, Gilbert A, Ramdharry G, Walumbe J, Shaw SE (2024). Video analysis of communication by physiotherapists and patients in video consultations: a qualitative study using conversation analysis. Physiotherapy.

[31] Wherton J, Shaw S, Papoutsi C, Seuren L, Greenhalgh T (2020). Guidance on the introduction and use of video consultations during COVID-19: important lessons from qualitative research. leader.

[32] Hutchings OR, Dearing C, Jagers D, Shaw MJ, Raffan F, Jones A, Taggart R, Sinclair T, Anderson T, Ritchie AG (2021). Virtual health care for community management of patients with COVID-19 in Australia: observational cohort study. J Med Internet Res.

[33] Yin RK (1984). Case Study Research: Design and Methods.

[34] Gill J, Johnson P (2002). Research Methods for Managers. Third Edition.

[35] Gerring J (2006). Case Study Research: Principles and Practices.

[36] Gummesson E (2000). Qualitative Research Methods in Management Research.

[37] Balfour M, Clarke C (2001). Searching for sustainable change. J Clin Nurs.

[38] Al-Busaidi ZQ (2008). Qualitative research and its uses in health care. Sultan Qaboos Univ Med J.

[39] Boeije H (2002). A purposeful approach to the constant comparative method in the analysis of qualitative interviews. Quality and Quantity.

[40] Kitzinger J (1995). Qualitative research. Introducing focus groups. BMJ.

[41] Litosseliti L (2003). Using Focus Groups in Research.

[42] Boyd H, McKernon S, Mullin B, Old A (2012). Improving healthcare through the use of co-design. N Z Med J.

[43] Yeates L, Gardner K, Do J, van den Heuvel L, Fleming G, Semsarian C, McEwen A, Adlard L, Ingles J (2022). Using codesign focus groups to develop an online community supporting families after sudden cardiac death (COPE-SCD) in the young. BMJ Open.

[44] Bertaux D, Kohli M (1984). The life story approach: a continental view. Annu. Rev. Sociol.

[45] Creswell JW (1998). Qualitative inquiry and research design: Choosing among five traditions.

[46] Guest G, Bunce A, Johnson L (2016). How many interviews are enough? An experiment with data saturation and variability. Field Methods.

[47] Morse JM (2016). Determining Sample Size. Qual Health Res.

[48] Mason M (2010). Sample size and saturation in PhD studies using qualitative interviews. Forum Qual. Soc. Res.

[49] Sandelowski M (2000). Whatever happened to qualitative description?. Res Nurs Health.

